# Supplementary material for: Functional genomics provide key insights to improve the diagnostic yield of hereditary ataxia
Source: Brain. 2023 Jan 10;146(7):2869–84. doi: 10.1093/brain/awad009 (PMC10316781; doi:10.1093/brain/awad009)
Supplement: awad009_Supplementary_Data [file awad009_supplementary_data.zip › brain-2022-01252-File009.pdf]

Supplementary Figures

Functional genomics provide key insights to improve the diagnostic yield of hereditary ataxia

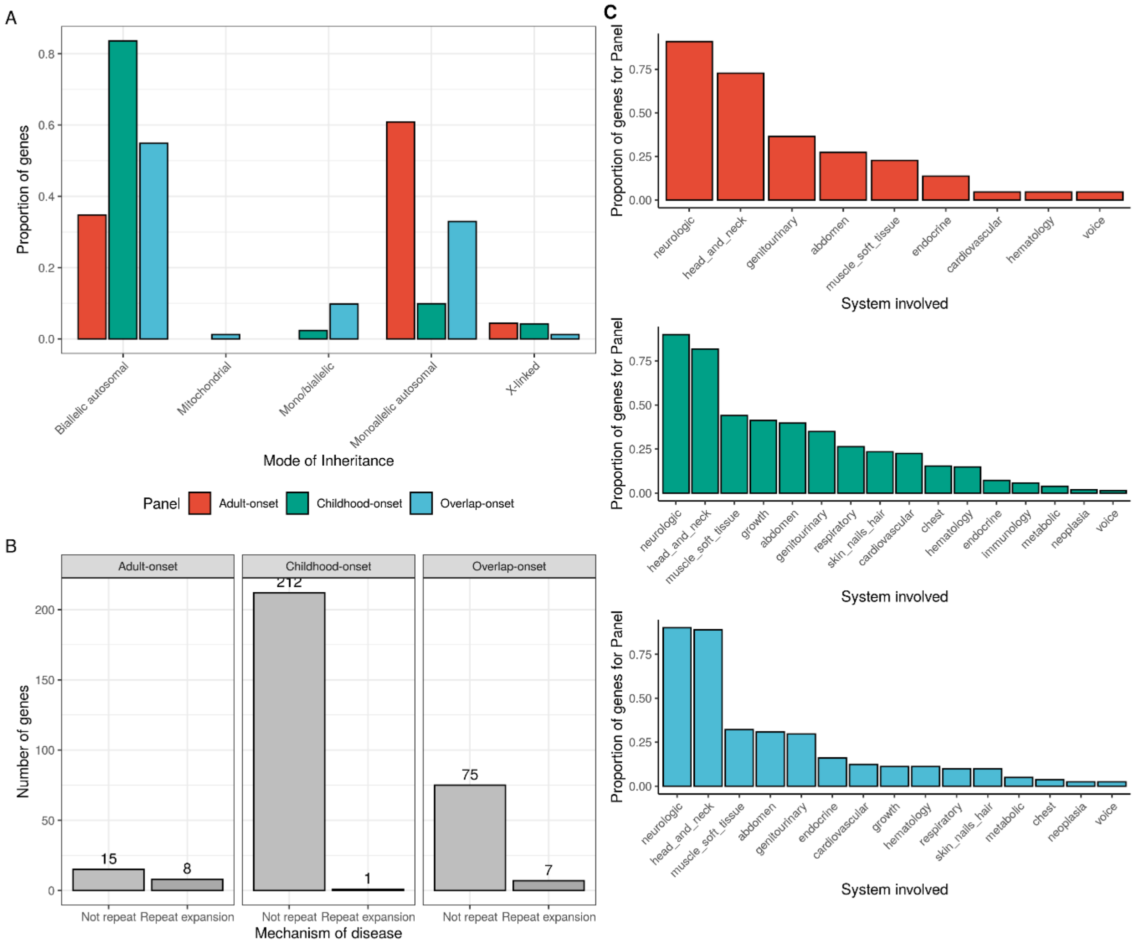

Supplementary Figure 1. Summary of the differences between genes known to cause ataxias using existing knowledge. (**A**) Proportion of genes with mode of inheritance across adult-onset, childhood-onset and overlap-onset hereditary ataxia genes. Definitions of mode of inheritance (per PanelApp) are as follows. Biallelic autosomal: A variant on both alleles of this gene is required to cause the disease; Mitochondrial: Variants in the gene in the mitochondrial genome are associated with disease; Mono/biallelic: The disease can be caused by a variant on one or both alleles of this gene; Monoallelic autosomal: A variant on one allele of this gene can cause the disease. X-linked: A variant in this gene can cause disease in males but may or may not cause disease in females. (**B**) Number of genes with known repeat expansions causing hereditary ataxia and number of genes not known to be associated with repeat expansion disorder. (**C**) Proportion of genes for that age-of-onset gene panel associated with disease in a particular system of the body as defined by OMIM across adult-, childhood- and overlap-onset ataxia genes. The corresponding horizontal lines on the boxplots represents the lowest quartile, median, and upper quartile of the data.

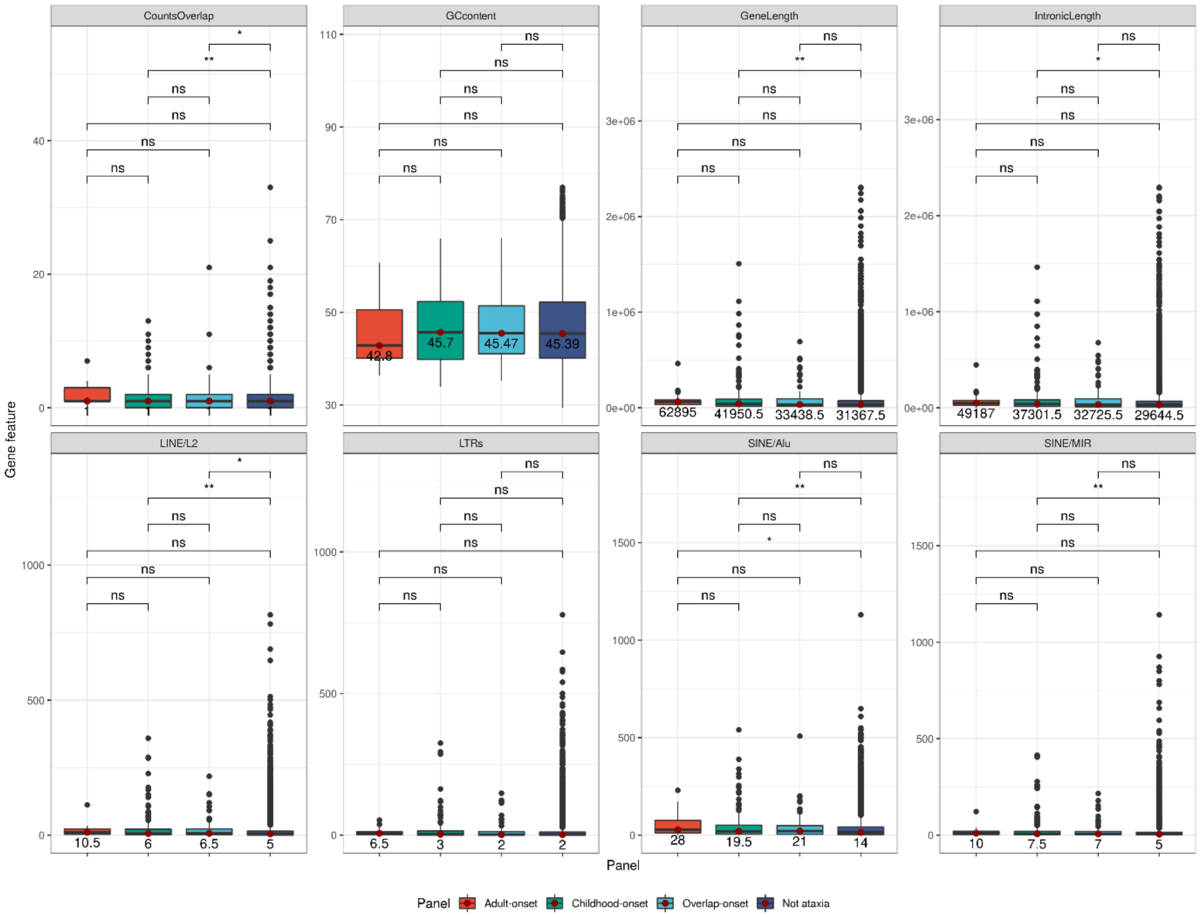

Supplementary Figure 2. Comparison of genic features capturing information about gene structure and gene complexity. CountsOverlap refers to the number of genes overlapping a gene of interest, GCcontent refers to the percentage GC content of a given gene, GeneLength is the length of each gene defined by its transcription start and end sites given in base pairs, IntronicLength is the total length of annotated introns within a gene. The bottom four panels shown the number of repeat elements per gene of LINE/L2 (long interspersed nuclear elements); LTRs (long terminal repeats), SINE/Alu (short interspersed nuclear elements/Alu sequences); SINE/MIR (SINE/mammalian-wide interspersed repeats) across the four different gene panels. Wilcoxon rank sum p-value comparing the gene lists are shown as follows: ns: $p > 0.05$; *: $p < 0.05$; **: $p < 0.01$; ***: $p < 0.001$; ****: $p < 0.0001$. The numbers below the red dots in the box and whisker plots represent the median values for that genic feature. The corresponding horizontal lines on the boxplots represents the lowest quartile, median, and upper quartile of the data. Further results are presented in Supplementary Table 5.

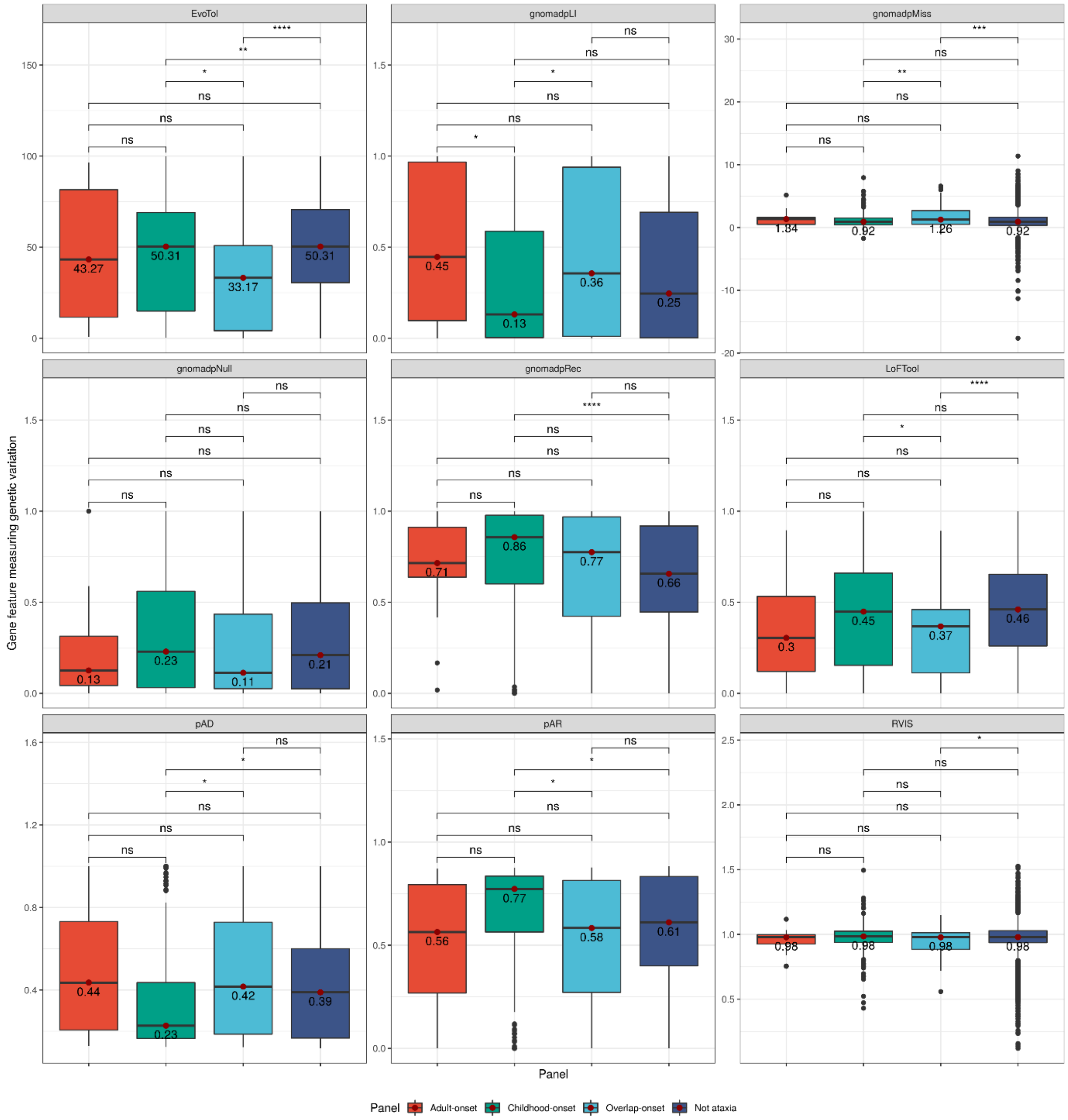

Supplementary Figure 3. Comparing genic measures of genic variation using data from publicly-available databases. LoFTool (gene intolerance score based on loss-of-function variants); EvoTol (quantifies a gene's intolerance to mutation using evolutionary conservation of protein sequences); RVIS (intolerance scoring system that assesses whether genes have more or less functional genetic variation than expected based on the apparently neutral variation found in the gene); pAD (probability of autosomal dominant variants); pAR (probability of autosomal recessive variants); gnomadpLI (loss-of-function score from gnomAD: pLI closer to 1 indicates that the gene or transcript cannot tolerate protein-truncating variation); gnomadpRec (probability of being intolerant of homozygous, but not heterozygous loss-of-function variants); gnomadpNull (probability of being intolerant to both homozygous and heterozygous variants); gnomadpMiss (probability of being intolerant of missense variants. Wilcoxon rank-sum p-value comparing the gene lists are shown as follows: ns: $p > 0.05$; *: $p < 0.05$; **: $p < 0.01$; ***: $p < 0.001$; ****: $p < 0.0001$. The numbers below the red dots in the box and

whisker plots represent the median values for that genic feature. The corresponding horizontal lines on the boxplots represents the lowest quartile, median, and upper quartile of the data. Further results are presented in Supplementary Table 5.

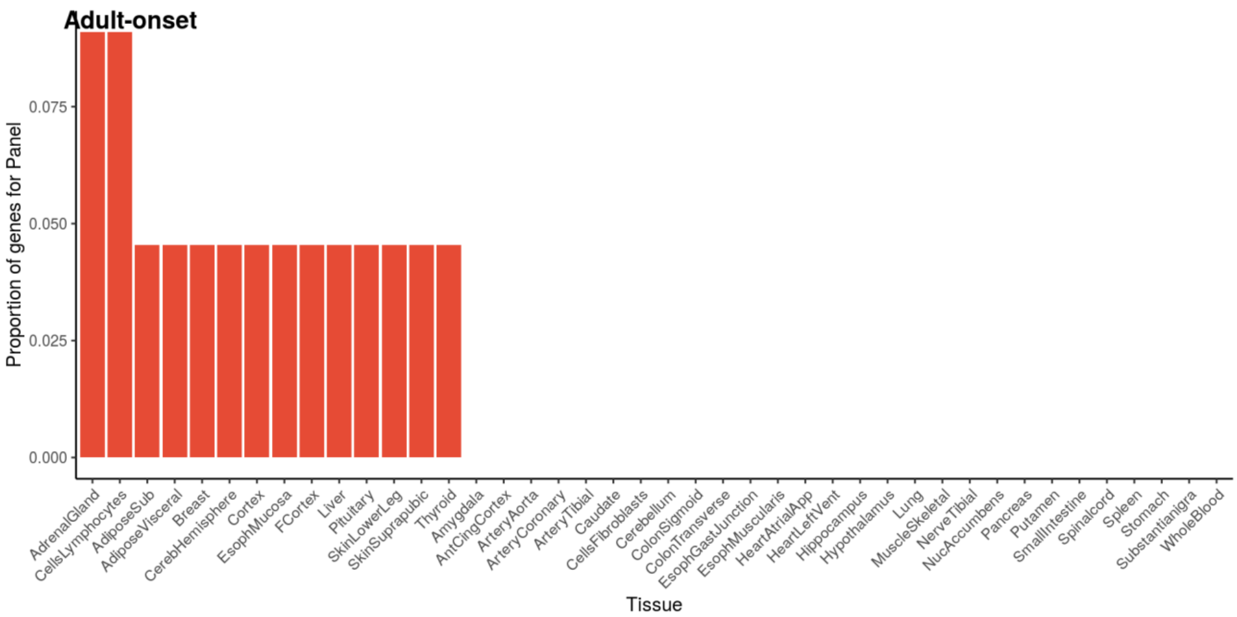
Adult-onset
Proportion of genes for Panel
0.000
0.025
0.050
0.075
AdrenalGland
CellsLymphocytes
AdiposeSub
AdiposeVisceral
Breast
CerebHemisphere
Cortex
EsophMucosa
FCortex
Liver
Pituitary
SkinLowerLeg
SkinSuprapubic
Thyroid
Amygdala
AntCingCortex
ArteryAorta
ArteryCoronary
ArteryTibial
Caudate
CellsFibroblasts
Cerebellum
ColonSigmoid
ColonTransverse
EsophGastJunction
EsophMuscularis
HeartAtrialApp
HeartLeftVent
Hippocampus
Hypothalamus
Lung
MuscleSkeletal
NerveTibial
NucAccumbens
Pancreas
Putamen
SmallIntestine
Spinalcord
Spleen
Stomach
Substantianigra
WholeBlood
Tissue

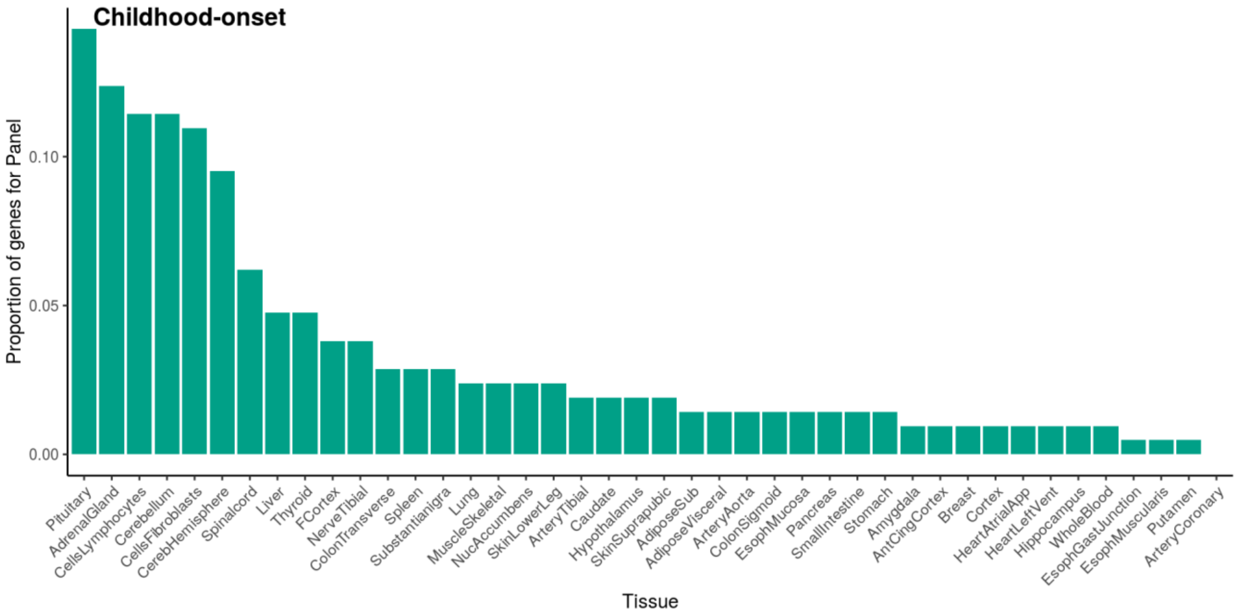
Childhood-onset
Proportion of genes for Panel
0.00
0.05
0.10
Pituitary
AdrenalGland
CellsLymphocytes
Cerebellum
CellsFibroblasts
CerebHemisphere
Spinalcord
Liver
Thyroid
FCortex
NerveTibial
ColonTransverse
Spleen
Substantianigra
Lung
MuscleSkeletal
NucAccumbens
SkinLowerLeg
ArteryTibial
Caudate
Hypothalamus
SkinSuprapubic
AdiposeSub
AdiposeVisceral
ArteryAorta
ColonSigmoid
EsophMucosa
Pancreas
SmallIntestine
Stomach
Amygdala
AntCingCortex
Breast
Cortex
HeartAtrialApp
HeartLeftVent
Hippocampus
WholeBlood
EsophGastJunction
EsophMuscularis
Putamen
ArteryCoronary
Tissue

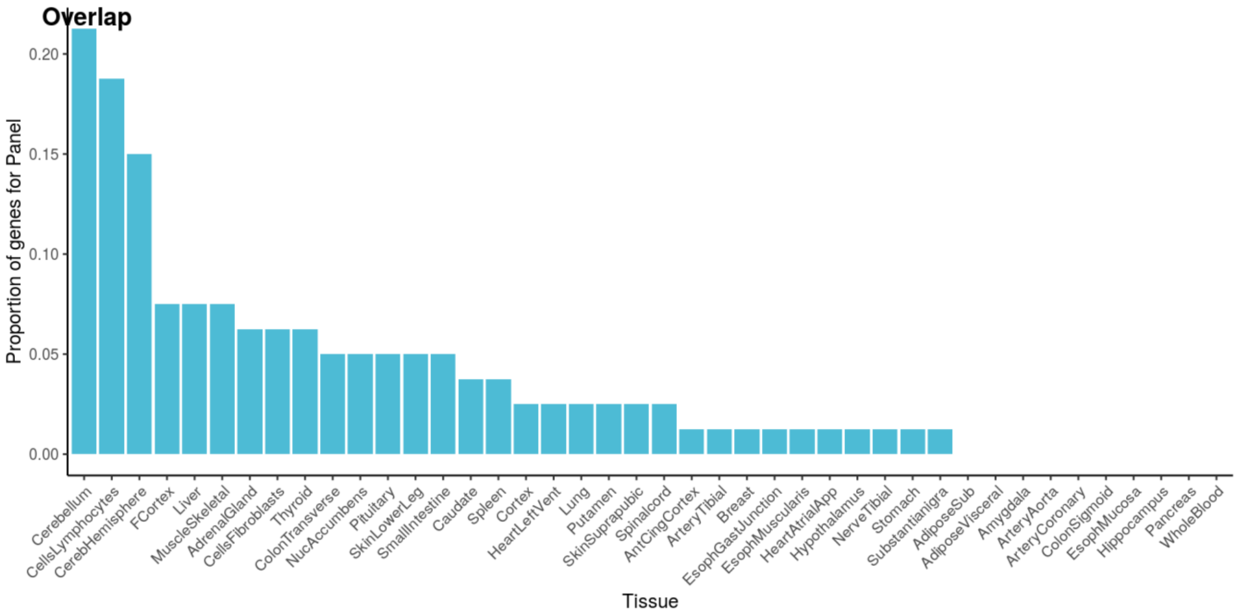
Overlap
Proportion of genes for Panel
0.00
0.05
0.10
0.15
0.20
Cerebellum
CellsLymphocytes
CerebHemisphere
FCortex
Liver
MuscleSkeletal
AdrenalGland
CellsFibroblasts
Thyroid
ColonTransverse
NucAccumbens
Pituitary
SkinLowerLeg
SmallIntestine
Caudate
Spleen
Cortex
HeartLeftVent
Lung
Putamen
SkinSuprapubic
Spinalcord
AntCingCortex
ArteryTibial
Breast
EsophGastJunction
EsophMuscularis
HeartAtrialApp
Hypothalamus
NerveTibial
Stomach
Substantianigra
AdiposeSub
AdiposeVisceral
Amygdala
ArteryAorta
ArteryCoronary
ColonSigmoid
EsophMucosa
Hippocampus
Pancreas
WholeBlood
Tissue

Supplementary Figure 4. Proportion of genes within each ataxia gene panel partitioned by age-of-onset with tissue-specific expression for each of the 47 Genotype-Tissue Expression Project (GTEx) tissues. Gene expression data were filtered for genes with >0.1 Reads Per Kilobase Million (RPKM) and corrected for batch effects, age, sex and RNA integrity number using ComBat. Residuals of these linear regression models were used to calculate tissue-specific expression. A gene was defined as having tissue-specific expression if expression in that tissue was five-fold higher than the mean across all tissues.

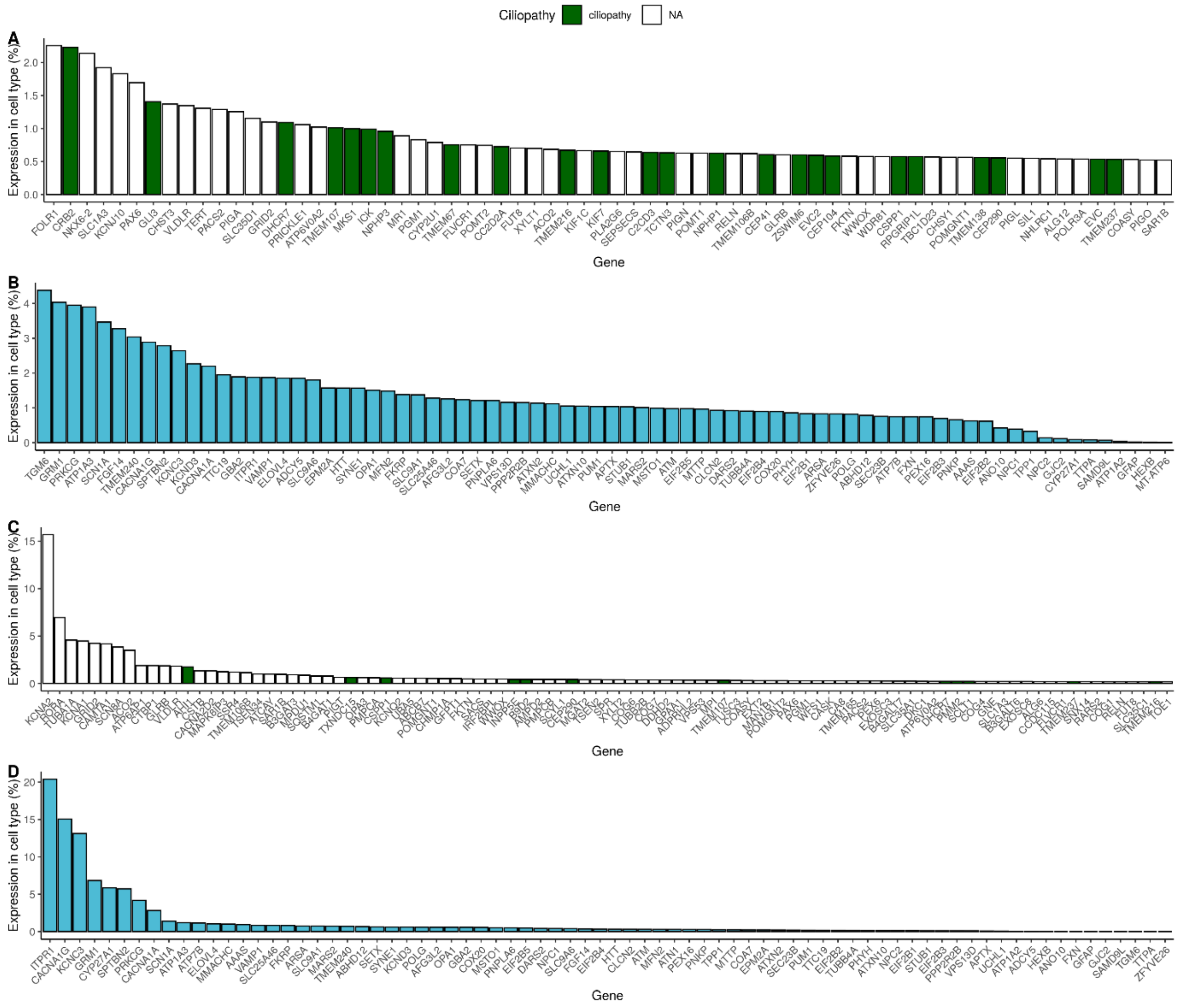

Supplementary Figure 5. Plot of specificity values for ataxia-associated genes. Specificity values were derived from Skene et al. (2018) who calculated specificity by dividing the mean expression of a gene in one cell type by the mean expression in all cell types. Specificity is the proportion of a gene's total expression attributable to one cell type, with a value of 0% meaning a gene is not expressed in that cell type and a value of 100% meaning that a gene is only expressed in that cell type. **(A)** Childhood-onset genes within CNS glia (level 2 cell types from the Karolinska single-cell RNA-sequencing) with ciliopathy genes shown in green; **(B)** Overlap-onset genes within CNS neurons (level 2 cell types); **(C)** Childhood-onset genes within cerebellar molecular interneurons (level 3 cell types from the Karolinska superset); (**D)** overlap-onset genes within cerebellar Purkinje cells (level 3).

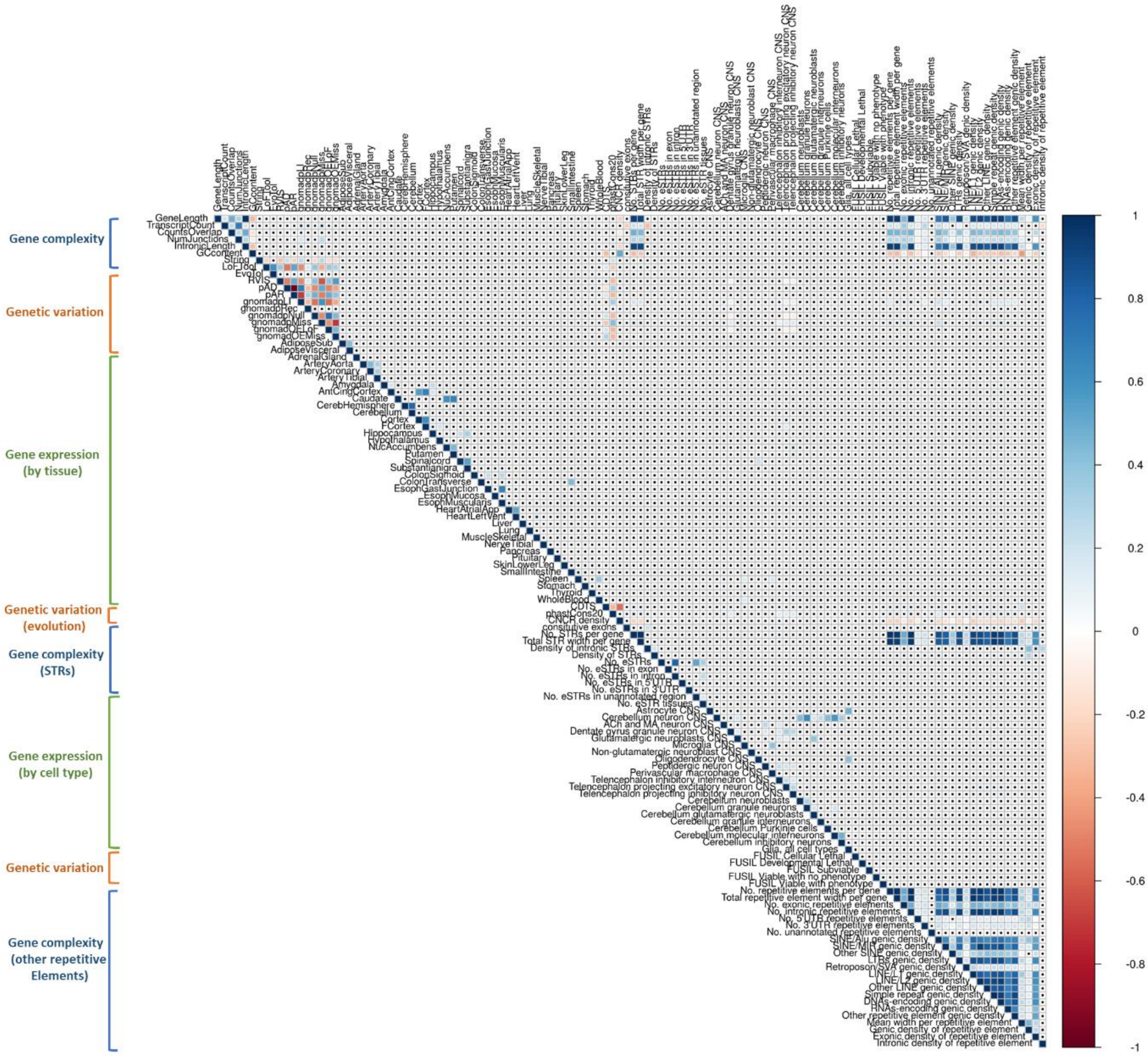

Supplementary Figure 6. Correlation matrix of the gene features classed by major groups of gene complexity, genetic variation and gene expression, by tissue and cell type. Pearson's correlation values are represented by the gradient shown. Module membership, adjacency, level 1 cell-type-specific expression are not shown in the plot for ease of data visualisation. Explanation for each individual feature is provided in Supplementary Table 2.

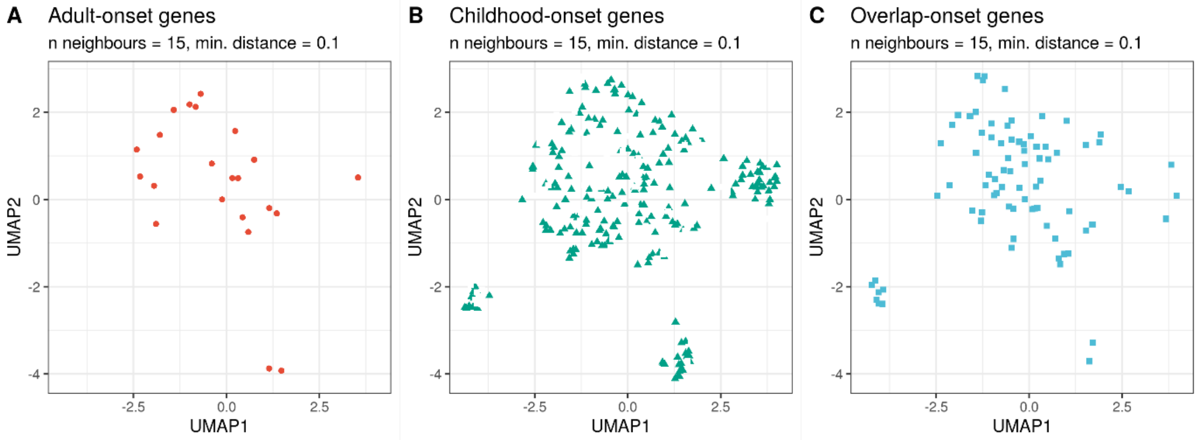

Supplementary Figure 7. Individual UMAP of ataxia genes partitioned by age-of-onset using 84 selected genic features from recursive feature elimination. (A) Adult-onset genes; (**B**) childhood-onset genes; (**C**) Overlap-onset genes.
