# Supplementary material for: Functional genomics provide key insights to improve the diagnostic yield of hereditary ataxia
Source: Brain. 2023 Jan 10;146(7):2869–84. doi: 10.1093/brain/awad009 (PMC10316781; doi:10.1093/brain/awad009)
Supplement: awad009_Supplementary_Data [file awad009_supplementary_data.zip › brain-2022-01252-File011.pdf]

Genomics England Research Consortium

Ambrose J. C. [1], Arumugam P.[1], Baple E. L. [1], Bleda M. [1], Boardman-Pretty F. [1,2], Boissiere J. M. [1], Boustred C. R. [1], Brittain H.[1], Caulfield M. J.[1,2], Chan G. C. [1], Craig C. E. H. [1], Daugherty L. C. [1], de Burca A. [1], Devereau, A. [1], Elgar G. [1,2], Foulger R. E. [1], Fowler T. [1], Furió-Tarí P. [1], Hackett J. M. [1], Halai D. [1], Hamblin A.[1], Henderson S.[1,2], Holman J. E. [1], Hubbard T. J. P. [1], Ibáñez K.[1,2], Jackson R. [1], Jones L. J. [1,2], Kasperaviciute D. [1,2], Kayikci M. [1], Lahnstein L. [1], Lawson K. [1], Leigh S. E. A. [1], Leong I. U. S. [1], Lopez F. J. [1], Maleady-Crowe F. [1], Mason J. [1], McDonagh E. M. [1,2], Moutsianas L. [1,2], Mueller M. [1,2], Murugaesu N. [1], Need A. C. [1,2], Odhams C. A.[1], Patch C. [1,2], Perez-Gil D. [1], Polychronopoulos D. [1], Pullinger J. [1], Rahim T. [1], Rendon A. [1], Riesgo-Ferreiro P.[1], Rogers T. [1], Ryten M. [1], Savage K. [1], Sawant K. [1], Scott R. H. [1], Siddiq A. [1], Sieghart A. [1], Smedley D. [1,2], Smith K. R. [1,2], Sosinsky A. [1,2], Spooner W. [1], Stevens H. E. [1], Stuckey A. [1], Sultana R. [1], Thomas E. R. A. [1,2], Thompson S. R. [1], Tregidgo C. [1], Tucci A. [1,2], Walsh E. [1], Watters, S. A. [1], Welland M. J. [1], Williams E. [1], Witkowska K. [1,2], Wood S. M. [1,2], Zarowiecki M.[1]
.

1. Genomics England, London, UK

2. William Harvey Research Institute, Queen Mary University of London, London, EC1M 6BQ, UK.
